# Long term outcomes of one-stage augmentation anterior urethroplasty: a systematic review and meta-analysis

**DOI:** 10.1590/S1677-5538.IBJU.2020.0242

**Published:** 2021-02-03

**Authors:** Cooper R. Benson, Gen Li, Steven B. Brandes

**Affiliations:** 1 Columbia University Medical Center Department of Urology New York NY USA Department of Urology, Columbia University Medical Center, New York, NY, USA; 2 Columbia University Medical Center Department of Biostatistics New York NY USA Department of Biostatistics, Columbia University Medical Center, New York, NY, USA

**Keywords:** Urethral Stricture, Systematic Review [Publication Type], Cystoscopy

## Abstract

**Objective:**

The objective is to summarize and characterize the long-term success of anterior augmentation urethroplasty (AU) in published series. The current literature on AU consists largely of retrospective series reporting intermediate follow-up and incompletely characterize the long term outcomes of AU.

**Materials and Methods:**

A systematic literature review was performed consistent with PRISMA guidelines to characterize long-term outcomes of AU with a minimum upper limit follow-up of 100 months. Penile/preputial skin flaps and graft and oral mucosal graft urethroplasties were included. The primary outcome was stricture-free survival for one-stage AU. Secondary analysis evaluated differences in outcomes based on two failure definitions: the need for intervention versus presence of recurrent stricture on cystoscopy or urethrography. Hazard rates were induced from the reported failure rates of one-stage AU and fixed and random effect models were fitted to the data. Additional subset analysis, removing potential confounders (lichen sclerosus, hypospadias and penile skin graft), was performed.

**Results:**

Ten studies met inclusion criteria, and two studies reported separate outcomes for grafts and flaps, and thus were included separately in the analysis. The mean hazard rate across all studies was 0.0044, the corresponding survival rates at 1 year 0.948, 5 years 0.766, 10 years 0.587, and 15 years 0.45. Subset analysis of the 4 select and homogeneous studies noted 1, 5, 10, and 15 years survival rates of 0.97, 0.96, 0.74, and 0.63, respectively.

**Conclusions:**

The long-term success rates of augmentation urethroplasty are appear to be worse than previously appreciated and patients should be counseled accordingly.

## INTRODUCTION

Urethroplasty is the gold standard treatment for urethral strictures, which is cost effective and has the highest reported success rates (1). There are a large variety of urethroplasty techniques that are utilized in modern practice. Nevertheless, regardless of the technique, the shared goals are of safety (minimal side effects), efficacy (unobstructed urethra with normal voiding), and durability (long-term effective results). Urethral reconstructive surgery decision making is highly nuanced and requires consideration of a multitude of factors, including stricture characteristics (length, location, and etiology), prior treatments, surgeon experience, co-morbidities and availability/quality of penile skin or graft material (2). Clearly, the surgical techniques and choice of graft/flap materials for augmentation urethroplasty (AU) have evolved over-time, and there is not a one-size fits all approach. Excision and primary anastomosis (EPA) urethroplasty has the highest reported durable success rates; (>85%); however, this is not always possible and often have to rely on buccal mucosal grafts (BMG) and/or penile skin flaps for successful reconstruction (3).

For AU, patients are often quoted an average success rate approximating 85% for augmentation urethroplasty. This is based on intermediate follow-up (∼5 years), with variable reported definitions of success (2). Moreover, the ICUD consensus statement on urethral strictures, prominently states that average success rates for one-stage bulbar urethroplasty is 83-88.8%, Asopa 86.7%, one-stage penile 75% and panurethral 88.2% (2, 4). While the durability of EPA urethroplasty is well documented, the long term success of AU has been poorly characterized. Thus, raising the question, is an 85% success rate an accurate representation of long-term AU success? We sought to characterize and analyze and summarize the long-term outcomes (10 and 15 year) of AU in literature. Our hypothesis is that the long-term outcomes of AU are well below the commonly quoted 85%.

## MATERIALS AND METHODS

### Search Strategy and Study Selection

A systemic literature review using Pubmed/MEDLINE and Embase databases from 2000-2018 was performed consistent with the Preferred Reporting Items for Systematic Reviews and Meta-Analyses (PRISMA) guidelines, in order to identify long-term outcomes and prognosis of augmentation urethroplasty (5). Terminology utilized for the data-base search included: Urethroplasty and long term outcomes, long term follow-up and urethroplasty, long term and urethroplasty, urethroplasty and outcomes, substitution urethroplasty, AU and outcomes, outcomes and urethral stricture. Inclusion criteria for meta-analysis included all studies reported in the adult literature on one-stage AU for anterior urethral strictures. Further inclusion criteria were studies that included patients who had follow-up of at least 100 months, and urethral reconstruction utilizing BMG, preputial/penile skin grafts and/or flaps. We included studies utilizing Palminteri urethroplasty, as well as Asopa inlay urethroplasty techniques (6, 7). The term “augmentation” urethroplasty was selected to encompass all of these techniques in accordance to the ICUD terminology consensus statement (8). We excluded studies and patients who underwent augmented anastomotic urethroplasty or staged urethroplasties, had the upper limit range of follow-up <100 months, and/or non-BMG and non-penile skin graft/ flap urethroplasties. Manuscripts without English translation were also excluded from the analysis as well as papers with incomplete or non-granular data.

**Figure 1:**
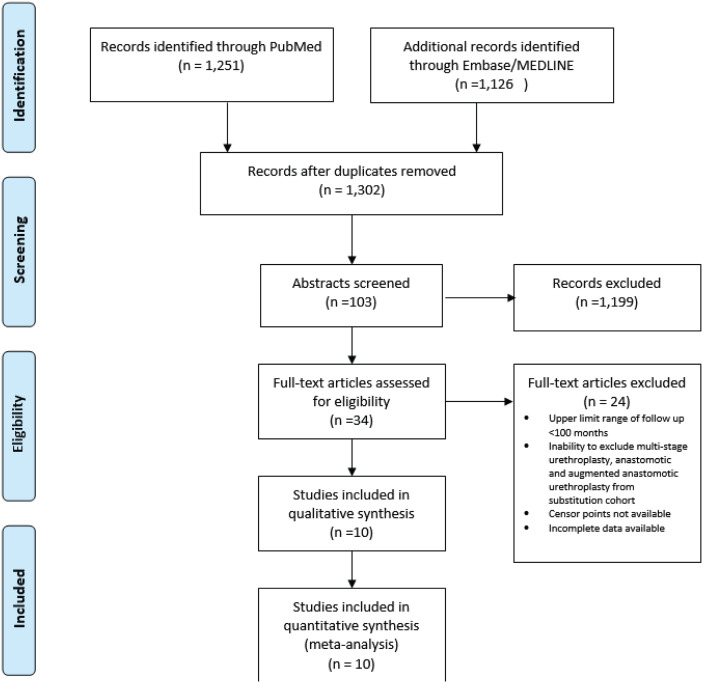
PRIMSA Diagram for inclusion and exclusion of studies in the meta-analysis.

Figure-1 demonstrates the PRISMA flow diagram and how studies were selected. We identified 1.302 unique articles from our search, of which 103 abstracts were reviewed, the remainder of the studies were excluded based largely on titles of the studies that were not relevant to the meta-analysis. Of the 103 abstracts reviewed in detail, only 34 abstracts included patients undergoing AU with adequate follow-up or unclear follow-up, based on abstract alone and these 34 manuscripts were reviewed by two authors (CRB, SBB). We identified 10 retrospective studies from expert urethral surgeon series, that met inclusion criteria, and classified as level 3 evidence.

### Data Extraction

When present, we extracted the following variables: number of patients, types of urethroplasty, substitution material, length of follow-up, definitions of success, Kaplan-Meier method censor points for stricture-free survival, failure rates, graft/flap location. Unpublished granular data was obtained from the author of one study (9) and was included in the analysis. Additionally, two studies reported outcomes of grafts and flaps separately, and thus were individually analyzed (9, 10). Several studies reported outcomes on non-AU one-stage reconstruction and these patients were excluded from analysis. If this was not possible because the lack of granularity, the entire study was excluded from analysis.

### Outcome Measures

The primary outcome was prognostic stricture free survival (SFS) of one-stage AU. All included studies did not utilize the same failure definition; 6 studies utilized the need for intervention following urethroplasty and 4 studies used presence of recurrent stricture on cystoscopy or urethrography during routine follow-up. Secondary analysis compared differences in SFS based on these definitions. An additional subset analysis was performed to eliminate potential confounders known to impact urethroplasty success, in an effort to create a “best case scenario.” In order to reduce contamination, we excluded patients and/or studies, when the data was not granular, that included patients with hypospadias and/or lichen sclerosus stricture etiologies, as well as the use of penile/preputial skin grafts.

#### Statistical analysis

The software package “metaphor” (Version 1.9-9) in R (Version 3.2.1) was used to conduct the meta-analysis on hazard rates. Hazard rates were induced from the reported failure rates of one-stage substitution anterior urethroplasty assuming survival followed an exponential distribution, and also using the above assumptions allowing us to combine the data. Random effects models were fitted to the data. Forrest plots were included to show effect sizes and confidence intervals for individual studies and also for the meta-analysis, as well as comparing the two definitions of failure. I2 analysis was used to represent heterogeneity among the studies and funnel plots were reported to represent the likelihood of publication bias in each analysis.

## RESULTS

**Figure 2:**
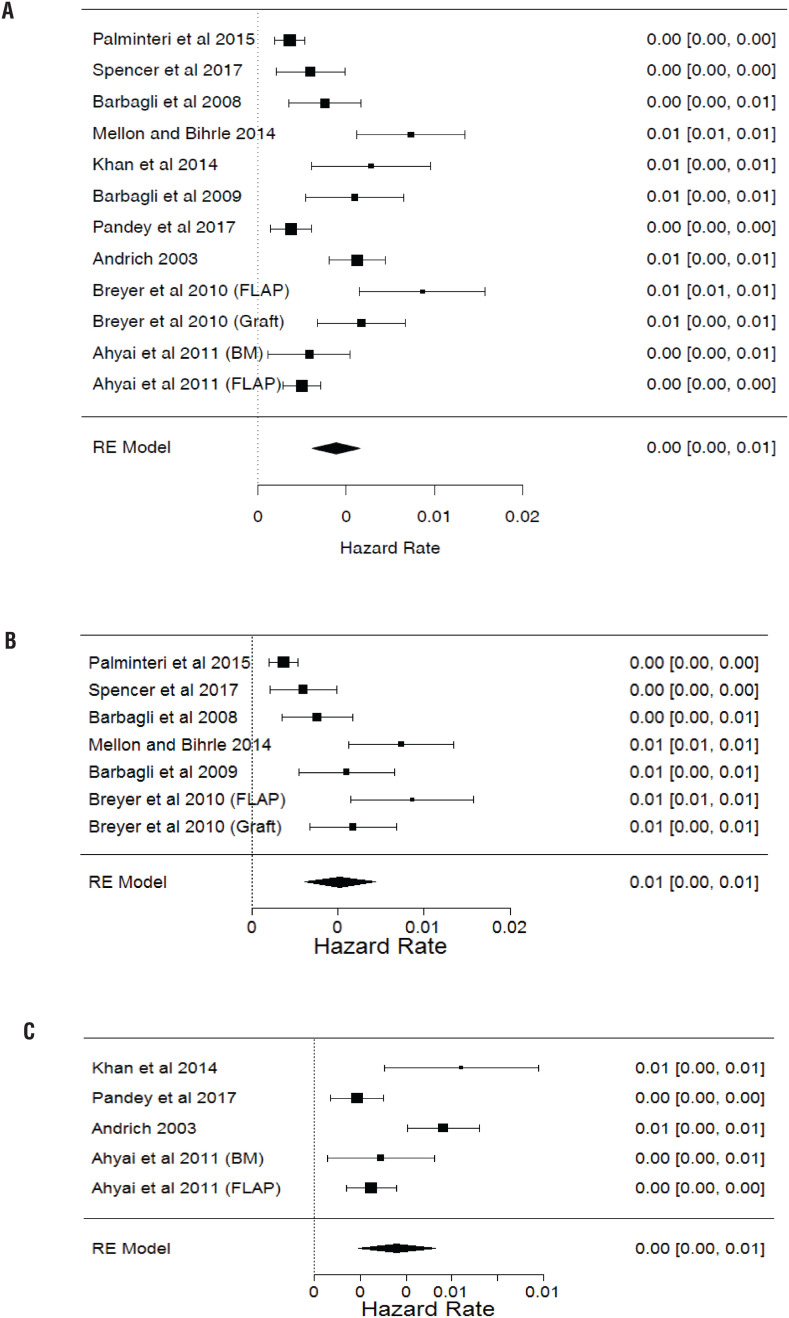
A) Forrest Plot of all studies included in analysis demonstrating the effect size of each study as hazard rates, which captures time to stricture recurrence in each individual study. B) Forrest Plot of Studies, demonstrating the effect size of each study using the need for instrumentation after urethroplasty as definition of failure. C) Forrest Plot of studies, demonstrating the effect size of each study, using recurrent stricture on routine urethrography or cystoscopy as definition of failure.

**Figure 3:**
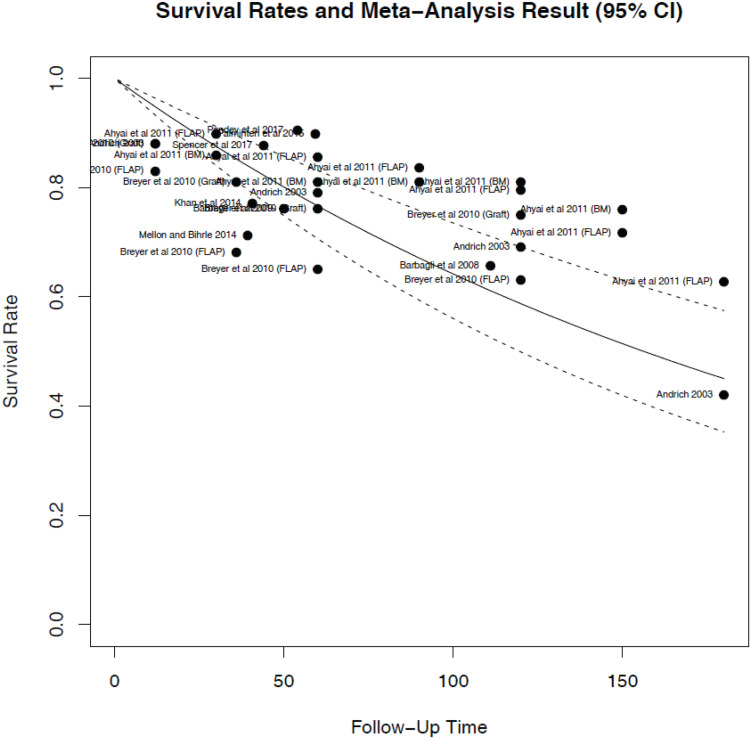
Estimated Kaplan-Meier curve overlay on the survival rates from each study included in the meta-analysis.

Supplementary Table-1 summarizes the granular data from these 10 studies (3, 6, 9–16). Across the studies, a total of 954 patients were included for analysis. Figure-2 A demonstrates the hazard rates of all included studies. The average hazard rate across all studies was 0.0044 (SD 0.000693), which corresponds to a 5-year survival rate of 0.766 (95% CI 0.706-0.831). Further, the survival rates at 1 year were 0.948 (95% CI 0.933-0.964), at 10 years, 0.587 (95% CI 0.499-0.691), and at 15 years, 0.45 (95% CI 0.352-0.574), as shown in the Kaplan-Meier curve in Figure-3. Two studies, Breyer et al. and Ahyai et al., reported separate outcomes for each fasciocutaneous skin flap and BMG patients, and thus were included separately in our analysis (9, 10).

**Supplementary Figure 1:**
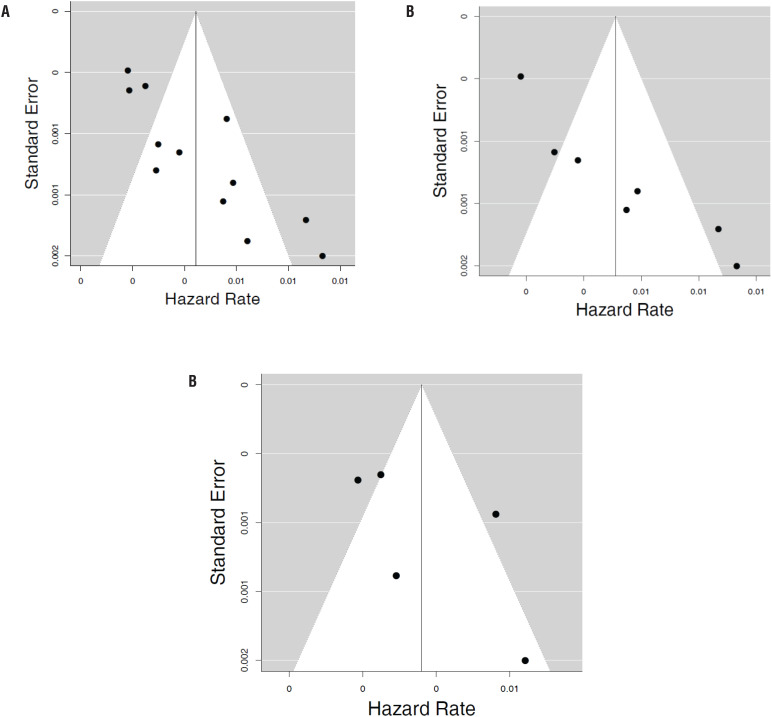
Funnel Plots indicating the presence of publication bias among the studies for (a) all included studies in the analysis (b) studies using the failure definition of need for instrumentation (c) studies using the failure definition of recurrent stricture on routine urethrography or cystoscopy.

The outcomes of urethroplasty were compared based on the definition of urethroplasty failure and found that the outcomes were not significantly different using either definition (p=0.132) (Figures 2B and 2C). We identified significant heterogeneity among the included studies in the meta-analysis (I2=85.66%, p <0.001). Additionally, we identified publication bias among the included studies all together and when divided by failure definition, which is demonstrated in the funnel plots in supplementary Figure-1.

**Figure 4:**
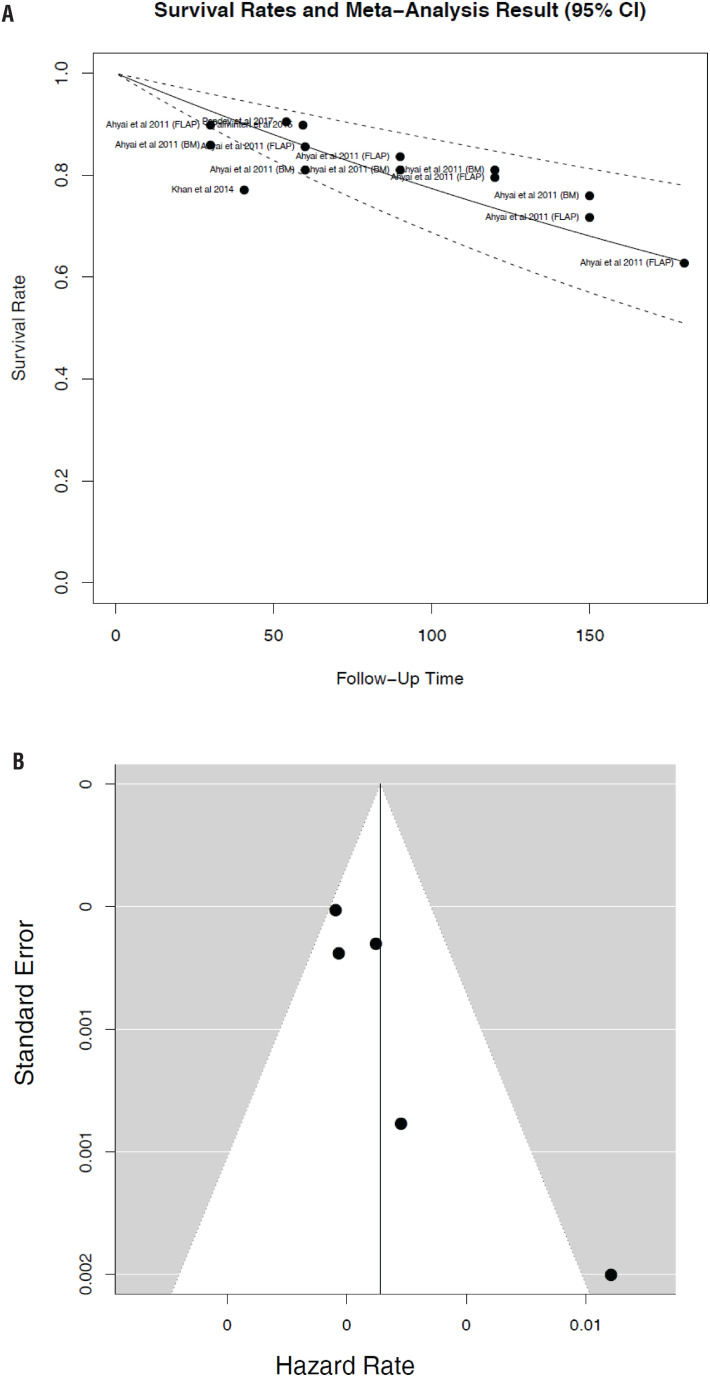
A) Sub-Analysis- Estimated Kaplan-Meier curve overlay on the survival rates from each study included in the meta-analysis, when excluding studies using penile skin grafts and reporting patients with a history hypospadias and lichen sclerosus. B) Funnel Plots of the same studies, indicating less publication bias than seen in the full analysis.

The subset analysis for the “best case scenario” included only four studies that met the criteria. Qualitative synthesis of the data from these four studies demonstrated corresponding survival rates 0.969 (95% CI 0.956-0.983) at 1 year, 0.857 (95% CI 0.798-0.920) at 5 years, 0.735 (95% CI 0.638-0.847) at 10 years, and 0.630 (95% CI 0.509-0.779) at 15 years (Figure-4A) and the corresponding funnel plot is shown in Figure-4B. There was a significant difference in SFS at all time points, when comparing these 4 “best case scenario” studies to the remaining 6 studies (p=0.0011).

## DISCUSSION

To our knowledge, this is the first study to combine expert series as a meta-analysis to evaluate the long-term success of AU. The current literature is deficient in reporting the long-term outcomes beyond five years for AU, largely low level of evidence from high volume expert surgeons and centers (2, 4). Certainly, it is well understood that at intermediate follow-up (5 years), urethroplasty success far exceeds that of endoscopic treatments. However, this data questions the long-term durability of these results for AU and suggests worse SFS than publish with intermediate follow-up.

In order to contextualize our results and meta-analyze the data, we relied on published outcomes of urethroplasty techniques that enabled us to combine the data for analysis. Vasudeva et al. randomized 80 patients to have either dorsal or ventral BMG onlay urethroplasty with non-inferior success rates, 92.5% versus 90% respectively (17). Similarly, Aldagadossi et al. randomized patients to dorsal onlay or inlay BMG urethroplasty and found no difference in success rates, 88% versus 86.4% (18). Furthermore, a recent systematic review also noted comparable success rates of 88% at 3 years, regardless of graft location (4). Thus, indicating that outcomes, regardless of BMG placement locations, are non-inferior. Additionally, there have been two randomized trials comparting dorsal BMG and penile skin flaps, demonstrating non-inferior success rates. Dubey et al. published success rates of 89.9% versus 85.6%, respectively, and similarly Soliman et al. found similar results with success rates of 89.5% versus 83.3% for BMG compared to penile skin flaps, respectively (19, 20). Lastly, there are several series demonstrating similar outcomes of free penile/preputial skin grafts AU compared to BMG AU. Alsikafi et al. reported similar success for penile skin graft (84%) and BMG AU (87%) (p=0.7375) (21). This was also corroborated by Raber et al., which found no significant difference in bulbar urethroplasty success comparing BMG and penile skin grafts (22). This is further supported by a systematic review, revealing recurrence rates of 14.5% and15.7% for grafts and flaps respectively (23). In contrast, a recent systematic review evaluated urethroplasty outcomes related to BMG versus penile skin grafts and found higher interim success of BMG (85.9%) compared to penile skin grafts (81.8%) (p=0.01) (24). However, our analysis was not powered to differentiate outcomes between penile skin/preputial flaps, penile skin/preputial grafts and BMG in urethral reconstruction. While objective and anatomic outcome measures are important, it is also essential to evaluate the quality of life and subject outcomes of urethral reconstruction.

Symptomatic urethral strictures interfere with voiding function and can have a profound impact on patients’ quality of life and the patient's family. The published definitions of success vary widely and include uroflowmetry, narrowing on cystourethroscopy or urethrography, calibration with bougienage, need for endoscopic intervention, as well as patient reported outcome measures (PROM) (1, 25). Bertrand et al. found that patient satisfaction based on PROM was strongly associated with objective measures of urethroplasty success (26). Moreover, there is a recent impetus to include PROMs as a measure of urethroplasty success, furthering the emphasis on patient satisfaction (27). The most common practice pattern among GURS members is to define urethroplasty failure as the “need for secondary procedures” and the most common methods for screening for stricture recurrence are uroflowmetry and post-void residual (25). However, invasive testing, such as routine cystoscopy (19%) and urethrography (17%), are less commonly utilized as routine screening methods by GURS members (25). Urethral surgery is mostly a quality of life surgery, and thus is successful if the patient reports improved quality of life and voiding function. This supports the notion that invasive testing is often overly aggressive and will not alter management, in the asymptomatic and satisfied patient.

Moreover, Baradaran et al. reported no difference in validated questionnaire scores between patients with normal cystoscopy (anatomic success), <17F and >17F recurrence of stricture (27). Among those with <17F recurrent stricture, 66% of the patients did not require secondary interventions, implying there was still subjective success and improved quality of life despite cystoscopic findings (27). Furthermore, our analysis demonstrated no significant difference in long-term outcomes comparing the definitions of success (p=0.132). This supports defining urethroplasty success, as the need for secondary intervention; however, the meta-analysis was not powered to answer such question.

As reconstructive urologists, patients are often quoted success rates of around 85% (75.6%-90.6%) for AU, which may be inflating its durability (2, 4). Furthermore, urethroplasty surgery is associated with a significant learning curve, between 100 and 400 cases, before achieving consistent successful outcomes (28, 29). Inexperienced urethral surgeons will likely not have the same results as the results in expert published series. A recent population analysis of urethroplasty outcomes, demonstrated a 32% failure rate for anterior urethroplasty at 3 years and trending toward 40% failure rate at 5 years, which highlights that community performed urethroplasty outcomes are worse than the 85% interim results by expert surgeons (30). The results of expert surgeon series are not necessarily reproducible by the occasional urethral surgeon. Thus, acknowledging this information is an essential aspect of the preoperative urethroplasty informed consent process. This analysis suggests a progressive deterioration rate over time for AU, which has been observed in multiple urethroplasty series (9, 10, 14, 31, 32). Several series have indicated that most failures occur within the first two years following urethroplasty; however, not all urethroplasty failures are equivalent (10, 14, 31). There are several potential explanations for AU failure; under-estimation of stricture length or degree of spongiofibrosis, failure of the graft to take due to poor host bed conditions, or flap loss due to compromised pedicle. This analysis is limited by lack of detail of failure patterns for AU, and thus are not able to incorporate this into our analysis.

We feel our conclusions about long-term success of AU are reliable even with the inherent limitations. After removing potential confounders in our subset analysis, the 15-year stricture free survival went from 45% to 63% for AU, which still falls short of the quoted 85% success at intermediate follow-up. Future studies would attempt to prospectively evaluate patients with similar stricture characteristics, risk factors, and surgeon experience and follow these patients’ long term. Furthermore, it is critical to better understand and identify the causes of progressive attrition in AU with long-term follow-up. Several potential causes of late failure include: an under-appreciation of the degree of spongiofibrosis at the time of reconstruction, progressive spongiofibrosis over time, and/ or subsequent urethral trauma leading to recurrence. Perhaps advanced imaging technology such as MRI or ultrasound elastography, will be enable more accurate characterization of spongiofibrosis that may not be readily apparent preoperatively or intraoperatively.

Our study limitations are merely a reflection of the lack of granularity of the reported outcomes of urethroplasty. The studies included herein are largely lower level of evidence and retrospective series with single surgeon or multiple expert surgeon series, and thus largely just observational and very heterogenous. Furthermore, as demonstrated in the funnel plots in supplemental Figure-1, there is publication bias which may have also impacted our results. Certainly, bias is also introduced with inclusion of a heterogeneous patient population with non-uniform stricture characteristics with variability in the AU grafts and/or flaps utilized and variable definitions of success.

## CONCLUSIONS

The long-term success of AU seems not as durable as reported with intermediate follow-up and appears to have continued deterioration with more than 100 months of follow-up. AU has worse than appreciated long term outcomes and further prospective studies are necessary to corroborate these findings. Our meta-analysis gives the urethral surgeon more information regarding the long-term success rates to aid in preoperative patient counseling. We cannot impart the long-term success of AU, unless we first acknowledge that long term results potentially are not as robust as once believed.

## APPENDIX

**Supplementary Table 1:** Summary statistics and analysis of the studies included for meta-analysis. This includes the study population, age, definition of failure, follow-up, and etiology.

Study	Study population	Mean Ages	Definition of Failure	Total Patients	Mean Follow up *Median	Failure Rate	Stricture Length Range	% with Prior Endoscopic Procedures	Number with prior urethroplasty	Etiology Breakdown	Number
		*Median			(Range)						
Andrich et al. 2003 (2)	Substitution Urethroplasty with penile/ preputial/scrotal onlay flap	47.2 years	Presence of stricture identified by symptoms and subsequent investigation	84	Not reported	Year (Rate)				Congenital/ hypospadias	7
					(10-15+ years)	1 (12%)				Infectious	--
						5 (21%)				Traumatic	--
						10 (31%)	--	--	--	Lichen Sclerosus	4
						15 (58%)				Idiopathic	16
										Iatrogenic	57
										Radiation	--
Barbagli et al. 2008 (12)	Bulbar Urethroplasty dorsal penile skin graft	43 years	Need for any intervention	38	111 months (80-149months)	(34.40%)	--	60.50%	0%	Congenital/ hypospadias	--
										Infectious	2
										Traumatic	7
										Lichen Sclerosus	--
										Idiopathic	18
										Iatrogenic	6
										Catheter	5
Barbagli et al. 2009 (15)	Penile Urethroplasty with orandi flap or Asopa inlay penile skin/OMG	51 years	Need for intervention	62	50 months (12-132 months)	24%		17.70%	4.80%	Congenital/ hypospadias	--
							---			Infectious	1
										Traumatic	4
							---			Lichen Sclerosus	--
										Idiopathic	19
										Iatrogenic	17
										Radiation	1
										Catheter	20
Breyer et al. 2010 (10)	Anterior Urethroplasty OMG (88 patients) Fasciocutaneous Flap (70 patients)	41 years*	Need for intervention	158	*69.6 months (1 month-120 months)	Year (Flap) ((Graft))				Congenital/ hypospadias	
						1 (17%) ((12%))				Infectious	--
						3 (32%) ((19%))				Traumatic	
						5 (35%) ((24%))	0.25cm-24cm	--		Lichen Sclerosus	--
						10 (37%) ((25%))				Idiopathic	--
										Iatrogenic	--
										Radiation	--
Ahyai et al. 2011, (Unpublished updated data presented 2014) (9)	Anterior urethroplasty OMG (22 patients) Flap (79 patients)	56 years	Presence of stricture on urethrogram, Uroflow < 15 cc/s	101	140 months (60-204 months)					Congenital/hypospadias	--
										Infectious	--
										Traumatic	--
						24.1% (OMG)	---	---	---	Lichen Sclerosus	--
						28.3% (Flap)				Idiopathic	--
										Iatrogenic	--
										Radiation	--
Mellon and Bihrle 2014 (13)	Ventral Buccal Graft Urethroplasty	42.4 years	Need for intervention	107	39.3 months (6.6-127.3 months)	28.90%		89%	11%	Congenital/hypospadias	--
										Infectious	5
										Traumatic	22
							1cm-8cm			Lichen Sclerosus	3
										Idiopathic	51
										Iatrogenic	26
										Radiation	--
Khan et al. 2014 (15)	Dorsal OMG Bulbar Urethroplasty	43.9 years	Stricture on Urethrogram	61	40.7 months (0113 months)	23%			0%	Congenital/hypospadias	--
										Infectious	--
										Traumatic	--
								---		Lichen Sclerosus	--
										Idiopathic	--
										Iatrogenic	--
										Radiation	--
Palminteri et al. 2015 (6)	Bulbar Urethroplasty Overlappying Dorsal plus ventral OMG	40 years	Need for intervention	166	*59.3 months (IQR 33-95.5 months)	10.20%		75%	1.20%	Congenital/hypospadias	--
										Infectious	--
										Traumatic	7
							1.9cm-3cm			Lichen Sclerosus	--
										Idiopathic	117
										Iatrogenic	8
										Catheter	34
Spencer et al. 2018 (11)	Anterior Urethroplasty dorsal onlay OMG, (Kulkarni) for long segment strictures	56 years	Need for intervention	73	44 months (12-162 months)	12.30%		92%	14%	Congenital/hypospadias	--
										Infectious	--
										Traumatic	8
							8cm-21cm			Lichen Sclerosus	28
										Idiopathic	18
										Iatrogenic	16
										Radiation	3
Pandey et al. 2017 (16)	Ventral Onlay OMG Urethroplasty	51 years*	Presence of Stricture on Cysto/ Urethrogram	104	*54 months (1-205 months)	9.60%	1cm-23cm		100%	Congenital/ hypospadias	--
										Infectious	--
										Traumatic	--
								________		Lichen Sclerosus	--
										Idiopathic	--
										Iatrogenic	--
										Radiation	--
